# Urinary bladder leiomyosarcoma with osteoclast-like multinucleated giant cells: a case report

**DOI:** 10.1186/s12885-019-5981-x

**Published:** 2019-08-02

**Authors:** Vincenzo Fiorentino, Francesco Pierconti, Niccolò Lenci, Martina Calicchia, Giuseppe Palermo, Pierfrancesco Bassi, Luigi Maria Larocca, Maurizio Martini

**Affiliations:** 1 Servizio di Istopatologia e Citodiagnosi, Fondazione Policlinico Universitario A. Gemelli IRCCS, Largo A. Gemelli 8, 00168 Rome, Italy; 2 Institute of Pathology, Università Cattolica del Sacro Cuore, Roma, Italy; 3 Institute of Urology, Università Cattolica del Sacro Cuore, Roma, Italy; 4 Clinica Urologica, Fondazione Policlinico Universitario A. Gemelli IRCCS, Largo A. Gemelli 8, 00168 Rome, Italy

**Keywords:** Leiomyosarcoma, Bladder, Osteoclast-like multinucleated giant cells

## Abstract

**Background:**

Bladder leiomyosarcoma is the most frequent mesenchymal neoplasm of the bladder. However, the rarity of the disease and some morphological aspects could give serious problems to differential diagnosis.

**Case presentation:**

A 86-year-old male patient was referred to our institution to undergo endoscopic low-urinary-tract re-evaluation 2 months after the detection of a “low-grade urothelial neoplasia” in urinary cytology. A TURBT (transurethral resection of bladder tumor) was performed and revealed a tumor extending for 3.5 cm with thin stalk peduncle on the left lateral wall of the bladder, cephalad and lateral to the left ureteral orifice. The exophytic part of the tumor was resected with the underlying bladder wall. Histologically, the tumor showed a quite complex pattern, composed of spindle cells, with often invasion to the surrounding bladder muscular wall, and the presence of numerous multinucleated, osteoclast-like giant cells, scattered throughout the neoplasia.

**Conclusions:**

Here we report a unique case of urinary bladder leiomyosarcoma with osteoclast-like multinucleated giant cells (OGCs). These cells, confounding the morphological aspect, indeed showed an immunohistochemical phenotype of non-neoplastic origin (most likely a histiocyte/macrophage differentiation). We feel that the presence of the OGCs within this tumor is reactive. Nevertheless, more research is necessary to understand the role of OGCs in urinary bladder tumors and leiomyosarcoma, in paticular.

## Background

In the United States, cancer of the urinary bladder is the fifth most common neoplasm, with 54,000 new diagnoses and 12,000 deaths every year [1]. The most important malignant bladder cancer is represented by urothelial cell carcinomas [2, 3]. Nonurothelial bladder neoplasms (including bladder sarcomas) represent 10% of bladder malignancies and constitute the most usual mesoderm-derived extraskeletal tumor of the bladder, where the leiomyosarcoma represents the most prevalent histological type. In literature, less than 200 cases of this tumor were described [3–6]. Rodriguez et al. reported the overall incidence of leiomyosarcoma of the bladder as approximately 0.23 cases per million [7]. Several studies proposed retinoblastoma (RB) gene mutations as a possible risk factor for this neoplasm, as well as the use of cyclophosphamide. The pelvic radiation therapy for other malignancies was also reported in literature as a possible risk factor [8]. Zhong et al. [6] have proposed the potential relationship between ketamine abuse and urinary bladder leiomyosarcoma describing a case of a young man with urinary bladder leiomyosarcoma, who had a history of chronic ketamine abuse: however, this relationship requires further scientific and clinical investigation.

The bladder leiomyosarcoma has a median age of incidence of 52 years and ranges widely from 16 to 83 years, with a higher incidence in women of reproductive age, which can suggest a possible role of the hormones in the pathogenesis of this neoplasm [3, 4, 9, 10]. When diagnosed, most of the neoplasms are in an advanced stage and less than 15% of tumors are identified in early stage. At macroscopic examination, they appear as submucosal nodules or ulcerating masses that can arise in any site of the bladder and they hardly affect the ureters or the renal pelvis, as opposed to urothelial neoplasms [4]. Grossly, it is often difficult to distinguish the tumor from a transitional or a squamous cell carcinoma. At microscopic examination, the typical features consist in interlacing fascicles of eosinophilic spindle cells with perinuclear vacuoles. Necrotic areas, anaplastic figures, high mitotic activity and epithelioid aspects may be present.

Patients’ clinical presentations typically involve dysuria, gross hematuria, or abdominal pain and patients may present with either severe obstructive voiding symptoms or obstructive uropathy depending on the size of tumor [11]. In literature, these neoplasms have generally been regarded as highly aggressive and associated with a poor prognosis. Higher survival rates have been achieved thanks to immediate radical cystectomy with wide margins and radical pelvic lymph node dissection. The main parameters to consider in order to better establish the prognosis are the size of the tumor, the degree of tissue involvement and the evaluation of margin involvement. Locally advanced disease needs neoadjuvant therapy, and the most commonly used regimen is doxorubicin, ifosfamide, cisplatin, adriamycin and vincristine with good outcomes [11]. According to Rodriguez et al. [7], the median overall survival is 46 months, the cancer-specific survival rates are 47% after 5 years from surgical resection and 35% after 10 years from surgical resection.

Overall local recurrence of these neoplasms is about 16%, with most recurrences located in the pelvis, and overall recurrence of distant metastases is about 53%, with the most common sites of metastases being the bone, lungs, brain and liver [8, 12].

## Case presentation

**Fig. 1:**
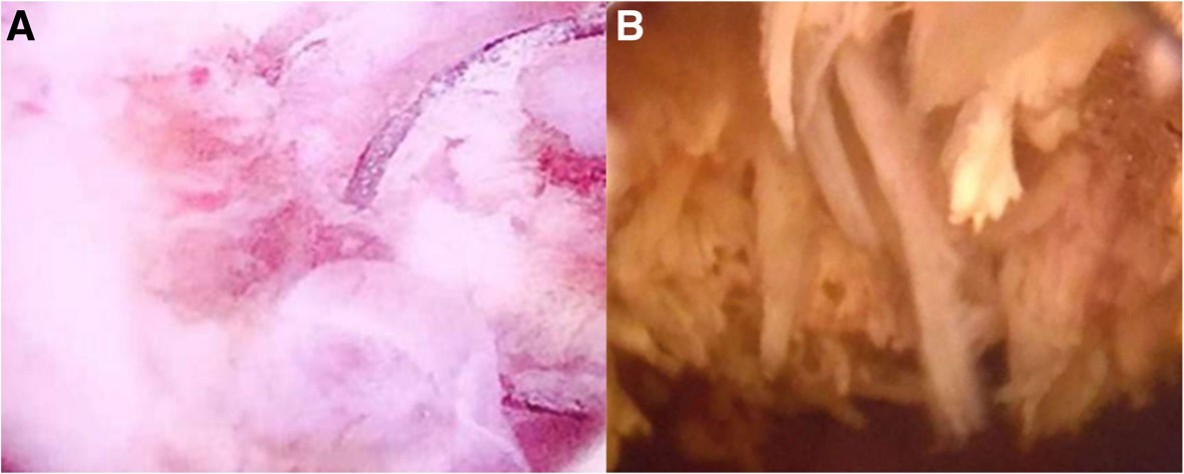
**a** and **b** panels show the cystoscopic images of the urinary bladder leiomyosarcoma

An 86-year-old male patient was referred to our institution in June 2017 to undergo an endoscopic low urinary-tract re-evaluation 2 months after the detection of a “low-grade urothelial neoplasia” in urinary cytology performed in an outside institution in a patient with hematuria. The patient had a history of non-muscle invasive bladder cancer (NMIBC) from 2002 (TaG2). The disease relapsed in 2007 and it was subsequently treated with Taxol + Hydat-b and intravescical BCG (Calmette-Guérin bacillus) until 2009. Follow up examinations were negative thereafter. A transurethral Resection of Prostate (TURP) was also performed in 2002 for benign prostate enlargement (BPE). A computed tomography (CT) scan of the abdomen and pelvis with intravenous contrast, performed at an outside institution 2 months prior, revealed numerous diverticula in the bladder in the absence of proliferative heteroformations. Past medical history highlighted a 30-year smoking history. The patient had hypertension, chronic kidney disease (creatinine clearance = 25 ml/min), and a sick sinus syndrome treated (2016) with a dual-chambered pacemaker implantation. Physical examination was negligible overall. An office cystoscopy, in February 2017, revealed a 2- to 3-cm tumor on the left lateral wall of the bladder (Fig. 1, panels a and b). A TURBT was performed in June 2017, and revealed a large tumor extended for 3.5 cm with a thin stalk peduncle on the left lateral wall of the bladder, cephalad and lateral to the left ureteral orifice. The exophytic part of the tumor was resected with the underlying bladder wall (July 2017). The remaining bladder quadrants were free of suspicious lesions even after NBI (Narrow Band Imaging) evaluation. The follow-up program included a cystoscopy, blood-analysis and whole-body CT scan every 6 months. In our case, the follow-up (most recently in January 2019) were negative, and the patient had no distant metastases.

### Histological and immunohistochemical features

**Fig. 2:**
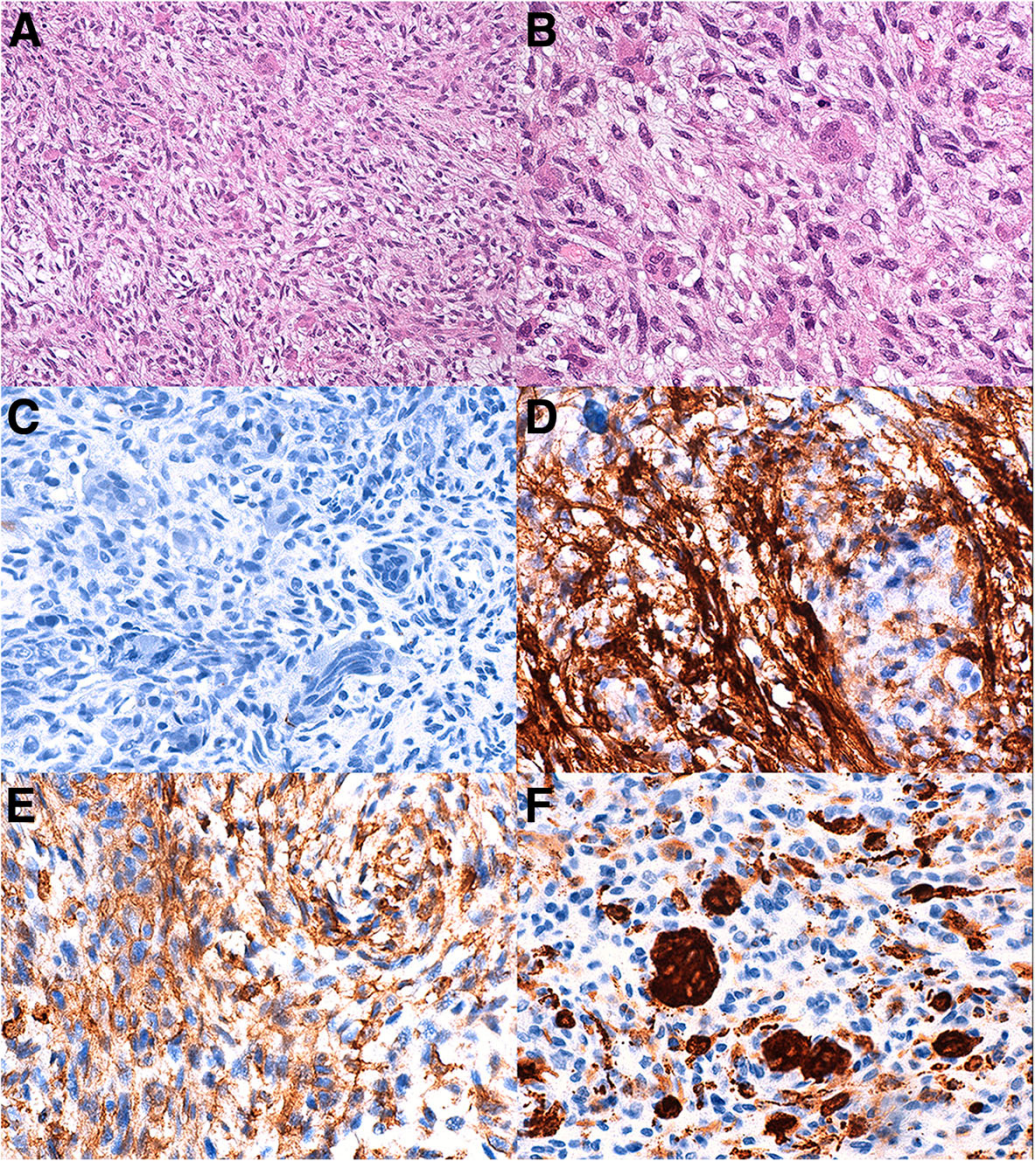
Histopathological findings of the urinary bladder leiomyosarcoma with osteoclast-like multinucleated giant cells. **a**, **b** Hematoxylin and eosin-stained (HE) shows a tumour composed of spindle cells with blunt-ended, cigar-shaped nuclei and copious eosinophilic cytoplasm without marked cellular atypia. The most striking feature is the presence of numerous multinucleated, osteoclast-like giant cells (OGCs), scattered throughout the neoplasm and, even when numerous, individually separated by the spindled cells of the leiomyosarcoma. (**A** HE 100×, **b** HE 400× magnification. **c** Tumor cells and OGCs staining negative for cytokeratin AE1/AE3 (400×). **d**, **e** Representative images for SMA (**d**, 400×) and CD44 (**e**, 400×). **f** OGCs staining positive for CD68 (400×))

The surgical specimens were formalin-fixed and paraffin embedded. The sections were stained with H&E. Immunohistochemistry was performed using avidin biotin complex technique and diaminobenzidine as chromogen. The immunohistochemistry was performed with vimentin (Dako, clone V9, monoclonal mouse anti-human), muscle actin (Dako, clone HHF35, monoclonal mouse anti-human), cytokeratin (Dako, clone AE1/ AE3, monoclonal mouse anti-human), CD44 (Dako, clone DF1485, monoclonal mouse anti-human), smooth muscle actin (Dako, clone 1A4, monoclonal mouse anti-human), CD68 (Dako, clone PG-M1, monoclonal mouse anti-human), Myoglobin (Dako, clone MYO7F7, monoclonal mouse anti-human), EMA (Dako, clone E29, monoclonal mouse anti-human), PSA (Dako, clone ER-PR8, monoclonal mouse anti-human), CK7 (Dako, clone OV-TL 12/30, monoclonal mouse anti-human), CK20 (Dako, clone KS20.8, monoclonal mouse anti-human), desmin (Dako, clone D33, monoclonal mouse anti-human), prostatic acid phosphatase (Dako, clone PASE/4LJ, monoclonal mouse anti-human), caldesmon (Dako, clone h-CD, monoclonal mouse anti-human), GATA-3 (Dako, clone L50–823, monoclonal mouse anti-human), high molecular weight cytokeratins (Roche-Ventana, clone 34ßE12, mouse monoclonal primary antibody), Ki-67 (Roche-Ventana, clone 30–9, rabbit monoclonal primary antibody), p63 (Roche-Ventana, clone 4A4, mouse monoclonal primary antibody), ALK (Dako, Clone ALK-1, M7195, mouse monoclonal antibody), ER (Dako, clone 1D5, mouse monoclonal antibody), PR (Dako, clone PgR636, mouse monoclonal antibody) and p53 (Roche-Ventana, clone Bp-53-11, mouse monoclonal primary antibody) following the manufacturer’s protocol dilution. We also performed routine positive and negative controls. Histologically, the tumor was composed of two components. The first component consists in interlacing fascicles of spindle cells with blunt-ended, cigar-shaped nuclei and copious eosinophilic cytoplasm without marked cellular atypia (Fig. 2a). The second component, sometimes prevalent, consisted of multinucleated, osteoclast-like giant cells (OGCs), dispersed throughout the tumor and, even when numerous, separated by the spindled cells of the leiomyosarcoma. The cytoplasm was finely granular and eosinophilic, and the nuclei were round or oval and often with a small round nucleolus (Fig. 2b). The first spindle cell component showed invasion of the surrounding bladder muscular wall, often areas of tumor necrosis and 4 mitoses per 10 high-power fields. There was neither mature bone nor osteoid tissue. The resection margins are free of tumor. Neoplastic cells were uniformly positive for vimentin and SM actin (Fig. 2d), focally for CD44 (Fig. 2e) and HHF35, negative for epithelial markers such as cytokeratin AE1/AE3 (Fig. 2c) and EMA. There was positivity for PR. The OGCs were positive for CD68 (Fig. 2f) and negative for ALK and epithelial markers such as cytokeratin AE1/AE3 (Fig. 2c) and EMA. These findings led us to a diagnosis of well differentiated bladder leiomyosarcoma with prominent osteoclast-type giant cell reaction (T1N0M0; G3, AJCC stage II).

## Discussion

Here we illustrate an unusual case of leiomyosarcoma with osteoclast-like giant cells arisen in the urinary bladder. We found no previous reports of leiomyosarcoma with osteoclast-like giant cells in this organ. In the bladder the OGCs have been described as osteoclast-rich undifferentiated carcinoma, a histological variant of urothelial carcinoma. The tumor is composed of mononuclear cells (frequently positive for epithelial markers), osteoclast-like giant cells (positive for CD51, CD68 and CD54) and an identifiable usual urothelial neoplasia (carcinoma in situ, papillary, or invasive carcinoma) in variable percentages. Some areas may resemble giant cell tumors of bone, while other areas may display single cells or aggregates of mononuclear cells with various degrees of atypia (including marked pleomorphism), different from the nuclei of OGCs. These mononuclear cells may be positive for EMA, pan-cytokeratin, CK7 and Cam5.2. Cytokeratin positivity, concomitant presence of high-grade urothelial neoplasia, matched p53 positivity in mononuclear cells and urothelial tumor cells, and poor prognosis denote this neoplasm as true undifferentiated carcinoma [13, 14]. Nevertheless, OGCs may be a non-specific finding and might be seen in low grade urothelial carcinomas. The presence of giant cells probably reflects a stromal response to the tumor [15] and is not related to prognosis. Another disease that sometimes presents a similar morphological aspect is the inflammatory myofibroblastic tumor. But in this case, the negativity for ALK1 and the absence of myxoid stroma are important points for a differential diagnosis. Ancient schwannoma could also be an entity with morphological similarities, but the negativity for S100 is the key point for a specific diagnosis. In our case, the positivity of OGCs for CD68 and the negativity for ALK, epithelial markers such as cytokeratin AE1/AE3 and EMA, could corroborate the hypothesis that OGCs only represent a stromal response to the presence of sarcomatous cells and don’t have a neoplastic nature.

OGCs have been described in a variety of extra-skeletal neoplasms, even if their presence is extremely rare: they have been documented in OGC carcinomas of the breast [16, 17] and pancreas [18], in mesenchymal tumors of different types [19, 20], in neoplasms from bladder [13, 21], thyroid [22], skin [23], ovary [24], adrenal [25], leiomyosarcomas of the uterus [26, 27] and in deep soft tissue [28].

The meaning of OGCs in tumors is not well understood and OGCs are generally thought to represent an unusual non-neoplastic tissue reaction [13, 21, 29]*.* According to recent analyses, neoplastic spindle cells of bone giant cell tumors may secrete a variety of cytokines and differentiation factors, including MCP1 (monocyte chemoattractant protein 1), ODF (osteoclast differentiation factor) and M-CSF (macrophage colony-stimulating factor): these are monocyte chemo-attractants and are essential for osteoclast differentiation. In this way, neoplastic cells could stimulate blood monocyte immigration into tumor tissues, enhancing their fusion into OGCs [30].

Cases of other malignant soft tissue tumors (also of the bladder) with the presence of OGCs are reported in literature [13, 15, 21, 31]. It appears that chemotactic factors expressed by some soft tissue sarcoma and carcinoma may be responsible for attracting and formation of these giant cells [15–29, 31]. There are no reported cases of leiomyosarcoma or other types of bladder sarcoma with osteoclastic cells and BCG instillation history. Notwithstanding, it has been shown that the formation of multinucleated giant cells and osteoclast fusion present a shared molecular signature. This suggest a common genetic bases [32] and it is presumable to think that the instillation of the BCG could also contribute to the pathogenesis of this type of reactive cells which were then further stimulated by sarcoma formation. However, further investigation and metanalysis are also required, especially in the area of epithelial bladder tumors.

## Conclusions

Urinary bladder leiomyosarcoma is an aggressive and rare malignant neoplasm. In this report, we presented a unique case of urinary bladder leiomyosarcoma with osteoclast-like multinucleated giant cells. This is the first case report describing OGCs in a urinary bladder leiomyosarcoma. The multinucleated giant cells observed in this case, confounding the morphological aspect and make the appropriate diagnosis difficult. We found that OGCs were characterized by a variable number of nuclei, including mononuclear forms. The general morphologic features suggested that these elements represented a part of the inflammatory and stromal response to the neoplastic spindle cells [16]. Immunohistochemical analyses confirmed that these cells are not neoplastic, but most likely histiocyte/macrophage derived cells. Nevertheless, more research is necessary to understand the role of OGCs in urinary bladder tumors in general and in leiomyosarcoma.

## Data Availability

The datasets used and/or analyzed during the current study are available from the corresponding author on reasonable request.

## Abbreviations

BCG: Bacillus Calmette-Guérin
BPE: Benign prostate enlargement
CT: Computed tomography
NBI: Narrow band imaging
NMIBC: Non-muscle invasive bladder cancer
OGCs: Osteoclast-like multinucleated giant cells
TURBT: Transurethral resection of bladder tumor
TURP: Transurethral resection of prostate
